# Meaningful nomograms based on systemic immune inflammation index predicted survival in metastatic pancreatic cancer patients receiving chemotherapy

**DOI:** 10.1002/cam4.7453

**Published:** 2024-07-10

**Authors:** Yanan Sun, Jiahe Hu, Rongfang Wang, Xinlian Du, Xiaoling Zhang, Jiaoting E, Shaoyue Zheng, Yuxin Zhou, Ruishu Mou, Xuedong Li, Hanbo Zhang, Ying Xu, Yuan Liao, Wenjie Jiang, Lijia Liu, Ruitao Wang, Jiuxin Zhu, Rui Xie

**Affiliations:** ^1^ Department of Digestive Internal Medicine Harbin Medical University Cancer Hospital Harbin Heilongjiang China; ^2^ Harbin Medical University Harbin Heilongjiang China; ^3^ Department of Internal Medicine Harbin Medical University Cancer Hospital Harbin Heilongjiang China; ^4^ Department of Pharmacology, College of Pharmacy Harbin Medical University Harbin Heilongjiang China

**Keywords:** chemotherapy, drinking, liver metastases, metastatic pancreatic cancer, nomogram, systemic immune–inflammation index

## Abstract

**Objective:**

The purpose of the study is to construct meaningful nomogram models according to the independent prognostic factor for metastatic pancreatic cancer receiving chemotherapy.

**Methods:**

This study is retrospective and consecutively included 143 patients from January 2013 to June 2021. The receiver operating characteristic (ROC) curve with the area under the curve (AUC) is utilized to determine the optimal cut‐off value. The Kaplan–Meier survival analysis, univariate and multivariable Cox regression analysis are exploited to identify the correlation of inflammatory biomarkers and clinicopathological features with survival. R software are run to construct nomograms based on independent risk factors to visualize survival. Nomogram model is examined using calibration curve and decision curve analysis (DCA).

**Results:**

The best cut‐off values of 966.71, 0.257, and 2.54 for the systemic immunological inflammation index (SII), monocyte‐to‐lymphocyte ratio (MLR), and neutrophil‐to‐lymphocyte ratio (NLR) were obtained by ROC analysis. Cox proportional‐hazards model revealed that baseline SII, history of drinking and metastasis sites were independent prognostic indices for survival. We established prognostic nomograms for primary endpoints of this study. The nomograms' predictive potential and clinical efficacy have been evaluated by calibration curves and DCA.

**Conclusion:**

We constructed nomograms based on independent prognostic factors, these models have promising applications in clinical practice to assist clinicians in personalizing the management of patients.

## 1 INTRODUCTION

Pancreatic cancer has the reputation of being the king of cancers, data released that China had 134,374 new pancreatic cancer patients and 131,203 pancreatic cancer deaths in 2022,^1^ and researches indicated that its prevalence is rising annually, with projections indicating that it will rank as the second most common cause of cancer‐related mortality by 2023.^2^, ^3^ When pancreatic cancer was initially detected, more than half of the patients developed metastases, the 5‐year survival rate for metastatic pancreatic cancer is fewer than 5%.^4^ Systemic chemotherapy^5^ has been proven to be the primary and preferred alternative for metastatic pancreatic cancer patients, the standard first‐line regimen is fluorouracil, leucovorin, irinotecan and oxaliplatin (FOLFIRINOX),^6^ nab‐paclitaxel and gemcitabine (AG),^7^ nab‐paclitaxel and S‐1 (AS),^8^ gemcitabine plus S‐1 (GS),^9^ and common clinical chemotherapy regimen is gemcitabine and oxaliplatin (GEMOX).^10^ However, in clinical practice, it has been found that patients have the heterogeneous survival prognosis. Therefore, personalized predictive models for metastatic pancreatic cancer patients receiving chemotherapy should be first developed and aim to be used in clinical decision making. Our study collected clinicopathological characteristics, laboratory blood test results and demographic information from patients in our hospital in order to build a prognostic model. Investigating the prognosis survival of patients with metastatic pancreatic cancer treated with first‐line chemotherapy is urgently needed, which is why this is being done. With this model, clinicians should be able to better customize treatment plans, which should increase patient survival.

Tumor growth and progression are significantly influenced by tumor microenvironment (TME),^11^, ^12^ the proliferation and invasion of tumor cells are facilitated by TME, extracellular matrix, blood vessels and mesenchymal cells are some of the components that give the tumor nourishment and oxygen while also forming the support structures that allow the tumor to grow. Furthermore, the TME's^13^, ^14^ signaling molecules and cytokines encourage tumor cells' ability to proliferate, migrate and invade new areas, tumor cells enter the blood or lymphatic system through blood vessels and mesenchymal cells in the TME and form metastases in other tissues and organs, and by interacting with immune cells, tumor cells can suppress their activity and evade the immune system's clearance. Furthermore, immune cell function can be inhibited by immunosuppressive substances and cells in TME, which lowers the immune response to tumors. In TME, cytokines and signaling pathways have the potential to increase tumor cell resistance to specific therapeutic agents and chemotherapeutic drugs.^15^ The inflammatory response may be one of the markers of tumor growth, which arises from the interaction of immune cells and tumor cells. Peripheral blood counts and TME are correlated,^16^ and TME contains a variety of immune cell types, including lymphocytes, monocytes, platelets and so on. Peripheral blood counts can be used to measure the quantity and activity of these immune cells. There are instances where a reduction in the TME's immune cell count causes a corresponding drop in the peripheral blood's immune cell count,^17^ white blood cell counts in peripheral blood can rise as a result of an inflammatory reaction in the TME. Because serum‐based biomarkers are non‐invasive, affordable and easily accessible, as well as having a high correlation with cancer patients' prognosis and individualization, they are becoming a more popular tool for prognostic estimation. Inflammatory statistics including platelet‐to‐lymphocyte ratio, systemic immunological inflammation index (SII), monocyte‐to‐lymphocyte ratio (MLR) and neutrophil‐to‐lymphocyte ratio (NLR), can now be used to assess the survival status of patients with pancreatic cancer. For instance, in 2015,^18^ it was demonstrated that patients undergoing pancreaticoduodenectomy who had a preoperative NLR ≥2.7 had a much poorer 2‐year survival than those whose NLR was <2.7. According to a prospective clinical trials,^19^ preoperative SII ≥873, indicates poorer outcomes for patients having pancreatic cancer resection, as opposed to NLR and platelet‐to‐lymphocyte ratio. Since no prior research has examined the predictive value of SII for overall survival (OS) and progression‐free survival (PFS) in patients with metastatic pancreatic cancer undergoing first‐line chemotherapy, the aim of our study is to assess the prognostic importance of SII in these patients.

Tumor prognostic models that are more user‐friendly and straightforward to use in clinical settings are constantly being developed thanks to technological advancements. Nomogram^20^ which are graphs made up of scales and a sequence of straight lines, are frequently used to quantify and show the extent to which certain factors have an impact on an outcome by charting the lines on the diagram. Typically, a nomogram is made up of scales and a collection of straight lines that combine to create a multidimensional environment. The scale shows the range of values for each line, which reflects an influential factor. The longer the line, generally speaking, the more significant the factor's influence on the outcome; including demographic data and clinicopathological features and inflammatory indicators derived from peripheral blood counts, independent prognostic factors^21^ were screened using Cox proportional risk regression models, then, numerous researchers have employed nomogram to visualize study results, facilitate clinical decision‐making and survival prediction, using R software reduces complex regression equations into understandable representations so that medical professionals can more readily comprehend and evaluate the predictive model outcomes. In order to visualize the OS and PFS of our patients, we built nomogram models based on SII, history of drinking and metastatic site.

## 2 MATERIALS AND METHODS

### 2.1 Patient information

This study is a retrospective clinical trial. By reviewing the case system of Harbin Medical University Cancer Hospital, 143 patients with metastatic pancreatic cancer who were treated with the clinically common two‐agent combination chemotherapy regimens of AG, AS, GS and GEMOX at Harbin Medical University Cancer Hospital from January 2013 to June 2021 were consecutively enrolled in this study. After the follow‐up period concluded in June 2023, patients who were released from the hospital had routine phone follow‐ups. Patients on a regular basis were gathered imaging data and clinical outcomes (survival, death or loss to follow‐up). Before receiving chemotherapy, all patients completed an informed consent form after learning about any potential side effects. Inclusion criteria: (1) patients were diagnosed with metastatic pancreatic cancer by pathological diagnosis or imaging examinations; (2) patients received first‐line chemotherapy regimens or clinically common chemotherapy regimens. Exclusion criteria: (1) patient received only one cycle of chemotherapy; (2) pMLRatients received blood transfusions before chemotherapy. Demographic and clinicopathological features were collected, including age, sex, primary site, metastasis sites, baseline carcinoembryonic antigen (CEA), baseline carbohydrate antigen 19‐9 (CA19‐9), the counts of peripheral neutrophils, monocytes, lymphocytes and platelets, body mass index (BMI), history of diabetes, history of smoking and history of drinking. Prior to chemotherapy, complete blood count and tumor markers were obtained within 7 days. In this study, widely used blood biomarkers such as SII, and NLR, were chosen for inclusion. MLR = monocyte count/lymphocyte count; the value of NLR was equal to the ratio of neutrophil count to lymphocyte count; SII = (neutrophil count*platelet count)/lymphocyte count. The Ethics Committee of the Harbin Medical University Cancer Hospital gave its approval and the study was carried out in compliance with the Declaration of Helsinki.

### 2.2 Clinical evaluation

The following was how we integrated our chemotherapy regimen in compliance with the standards and our clinical practice: (1) AG; (2) AS; (3) GS; (4) GEMOX. Gemcitabine (1000 mg/m^2^) d1, d8 intravenously and albumin‐bound paclitaxel (125 mg/m^2^) intravenously, and 3 weeks for 1 cycle of chemotherapy were administered to patients in the AG group; S‐1 d1‐d14 orally and albumin‐bound paclitaxel (125 mg/m^2^)intravenously, and 3 weeks for 1 cycle of chemotherapy were administered to patients in the AS group; gemcitabine (1000 mg/m^2^) d1, d8 intravenously and S‐1 d1‐d14 orally, and 3 weeks for 1 cycle of chemotherapy were administered to patients in the GS group; patients in the GEMOX group received gemcitabine (1000 mg/m^2^) d1, d8 intravenously and oxaliplatin (130 mg/m^2^) d1 intravenously. The dosage of S‐1 was based on body surface area; for body surface areas higher than 1.5 m^2^, higher than 1.25 m^2^ and less than 1.5 m^2^,and less than 1.25m^2^, it was 120 mg/day, 100 mg/day or 80 mg/day, respectively. Primary endpoints were OS and PSF; the disease control rate (DCR), adverse effects and objective response rate (ORR) were indications of secondary study endpoints. The duration between a patient's first diagnosis and death was defined in the current study as OS, and the time between a patient's initial diagnosis and either tumor progression or death was defined as PFS. Patients with metastatic pancreatic cancer had their treatment responses evaluated using the Response Evaluation Criteria in Solid Tumors (RECIST) 1.1. Utilizing the Common Terminology Criteria for Adverse Events (CTCAE), patients' AEs were evaluated.

### 2.3 Statistical analysis

Excel was utilized for data management, IBM SPSS Statistics 27 was employed for analyzing the data, and GraphPad Prism 9 and R (version 4.1.1) were utilized for data visualization in the analysis. The measurements were reported as mean ± standard deviation for a normal distribution and as median (interquartile range, IQR) for an abnormal distribution. The expression for count data was a constant (rate) (*n* [%]). The receiver operating characteristic (ROC) with area under the curve (AUC) was used for the identification of the optimal cut‐off value. The Kaplan–Meier survival analysis method with log rank test was utilized to analyze the correlation between patients' survival and the variable. Cox regression analysis was applied to screen for independent prognostic factors of OS and PFS. The “rms” package in R software was run to plot nomograms, calibration curve was a regression model to assess the consistency of novel models. Models' net benefit was evaluated using decision curve analysis (DCA). *P* < 0.05 represents statistical significance.

## 3 RESULT

### 3.1 Patient characteristics

**FIGURE 1:**
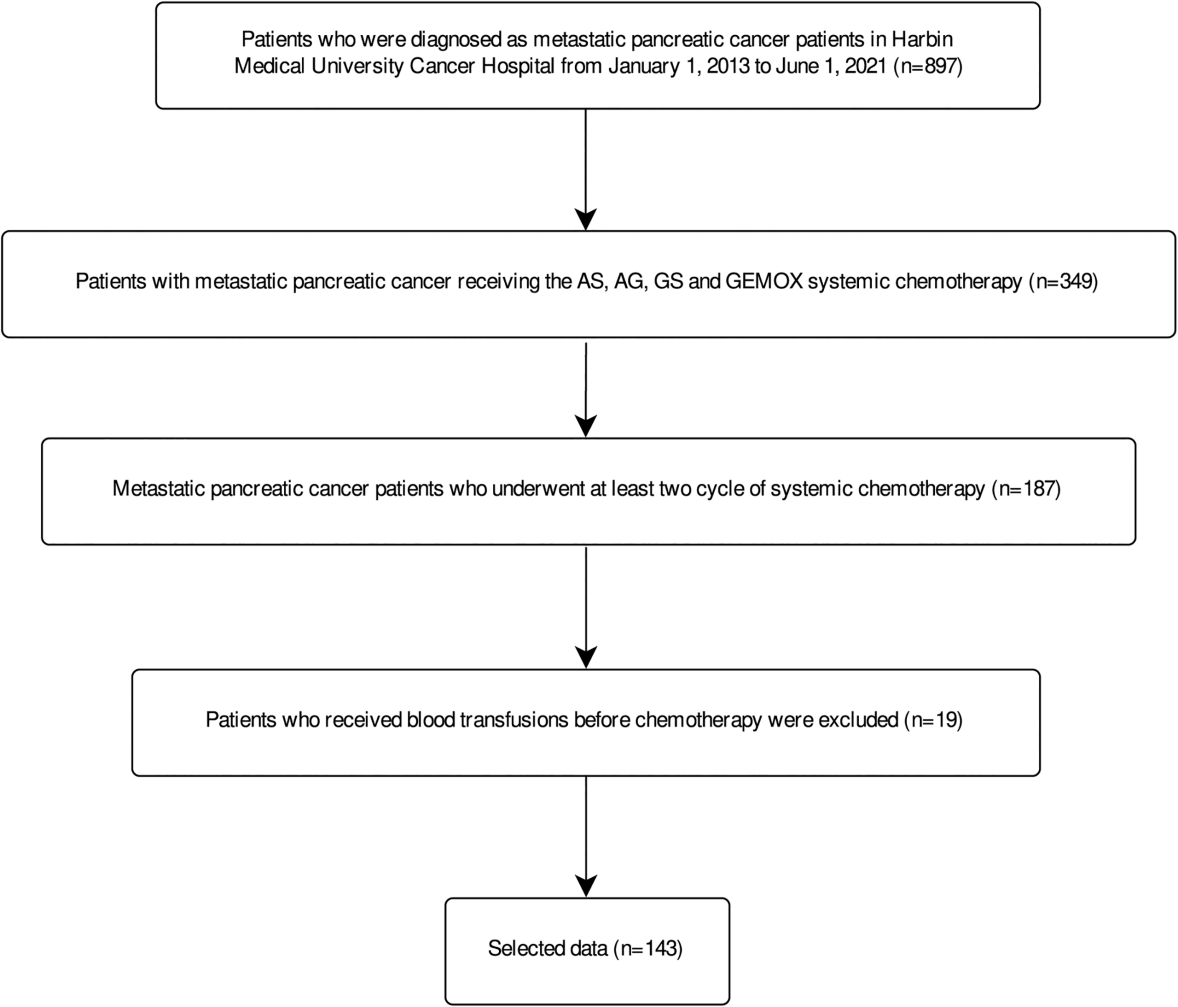
Patients enrollment process.

We pre‐included 897 patients who received a metastatic pancreatic cancer diagnosis at the Harbin Medical University Cancer Hospital between January 2013 and June 2021. We screened 349 patients who received chemotherapy with AS, AG, GS, and GEMOX regimens, 187 patients who received two or more cycles of chemotherapy, 19 patients who received blood transfusions during chemotherapy. Ultimately, our analysis comprised 143 patients with metastatic pancreatic cancer (Figure 1).

Table 1 showed the initial characteristics of each patient, such as age, sex, primary site, metastasis sites, baseline CA19‐9, baseline CEA, baseline SII, baseline MLR, baseline NLR, BMI, history of diabetes, history of smoking, history of drinking and systemic chemotherapy regimen. Of the 143 patients, 48 were older than 60 years old, making up 33.5% of the total; 59 patients were female, making up 41.2% of the total; the majority of tumor primary sites were in the pancreatic head; liver metastases accounted for the majority of tumor metastases, making up approximately 70% of the total number of patients; only 81 patients had elevated CEA, and 127 patients had elevated CA19‐9. Twelve patients (8.4%) had a BMI of less than 18.5, while 33 patients (23.1%) had a BMI of more than 25. Additionally, 17 patients (11.9%) had a history of diabetes, 28 patients (19.6%) had a history of smoking, and 18 patients (12.6%) had a history of drinking.

**TABLE 1:** The baseline characteristics of patients.

Clinical data	*n* (%)
Age	
≤60	95 (66.5)
>60	48 (33.5)
Sex	
Women	59 (41.3)
Men	84 (58.7)
Primary site	
Head	47 (32.9)
Neck	8 (5.6)
Body	21 (14.7)
Tail	34 (23.8)
Head, Body	4 (2.8)
Neck, Body	2 (1.4)
Body, Tail	27 (18.9)
Metastasis sites	
Liver	100 (69.9)
Other	43 (30.1)
Baseline CA19‐9	
<37	12 (8.4)
≥37	127 (88.8)
Unknow	4 (2.8)
Baseline CEA	
<5	55 (38.5)
≥5	81 (56.6)
Unknow	7 (4.9)
Baseline SII	
<966.71	98 (68.5)
≥966.71	45 (31.5)
Baseline MLR	
<0.275	59 (41.3)
≥0.275	84 (58.7)
Baseline NLR	
<2.54	50 (35.0)
≥2.54	93 (65.0)
BMI	
<18.5	12 (8.4)
18.5–24.9	98 (68.5)
25.0–30.0	28 (19.6)
>30	5 (3.5)
History of diabetes	
No	126 (88.1)
Yes	17 (11.9)
History of smoking	
No	115 (80.4)
Yes	28 (19.6)
History of drinking	
No	125 (87.4)
Yes	18 (12.6)
Chemotherapy regimen	
AS	45 (31)
AG	28 (20)
GS	39 (27)
GEMOX	31 (22)

Abbreviations: AG, nab‐paclitaxel and gemcitabine; AS, nab‐paclitaxel and S‐1; BMI, body mass index; CA19‐9, Carbohydrate antigen19‐9; CEA, carcinoembryonic antigen; GEMOX, gemcitabine and oxaliplatin; GS, gemcitabine plus S‐1; MLR, monocyte‐to‐lymphocyte ratio; NLR, neutrophil‐to‐lymphocyte ratio; SII, systemic immune inflammation index.

### 3.2 Survival Estimates

**FIGURE 2:**
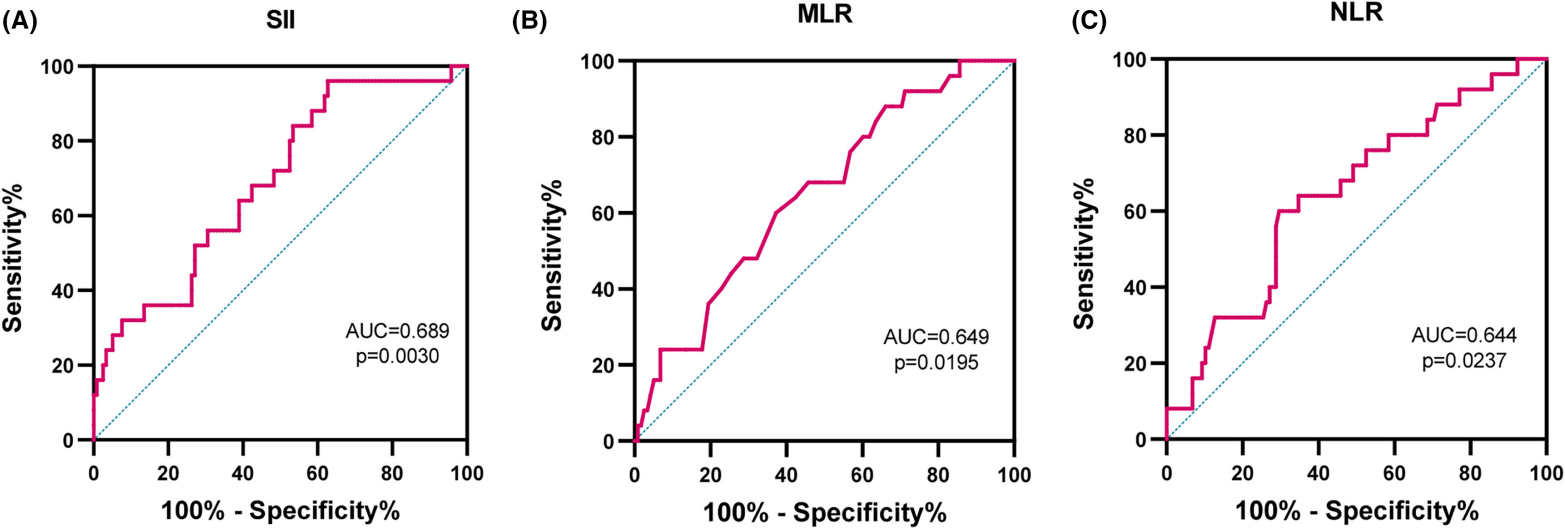
The ROC curve with AUC of SII (A), MLR (B) and NLR (C), *p* < 0.05 is considered statistically significant.

We studied the survival of all patients at 2 years after diagnosis, the mOS of patients was 9.7 months. And 7 patients (4.8%) were lost to follow‐up and 118 of the 143 patients (82.5%) had died. ROC analysis and AUC determined the optimal SII, NLR and MLR cutoff values of 966.71, 2.54 and 0.257, respectively (*p* < 0.05) (Figure 2), the sensitivity and specificity were found to be statistically significant (*p* < 0.05) at 0.373 and 0.96, 0.703 and 0.6, and 0.627 and 0.6, respectively.

**FIGURE 3:**
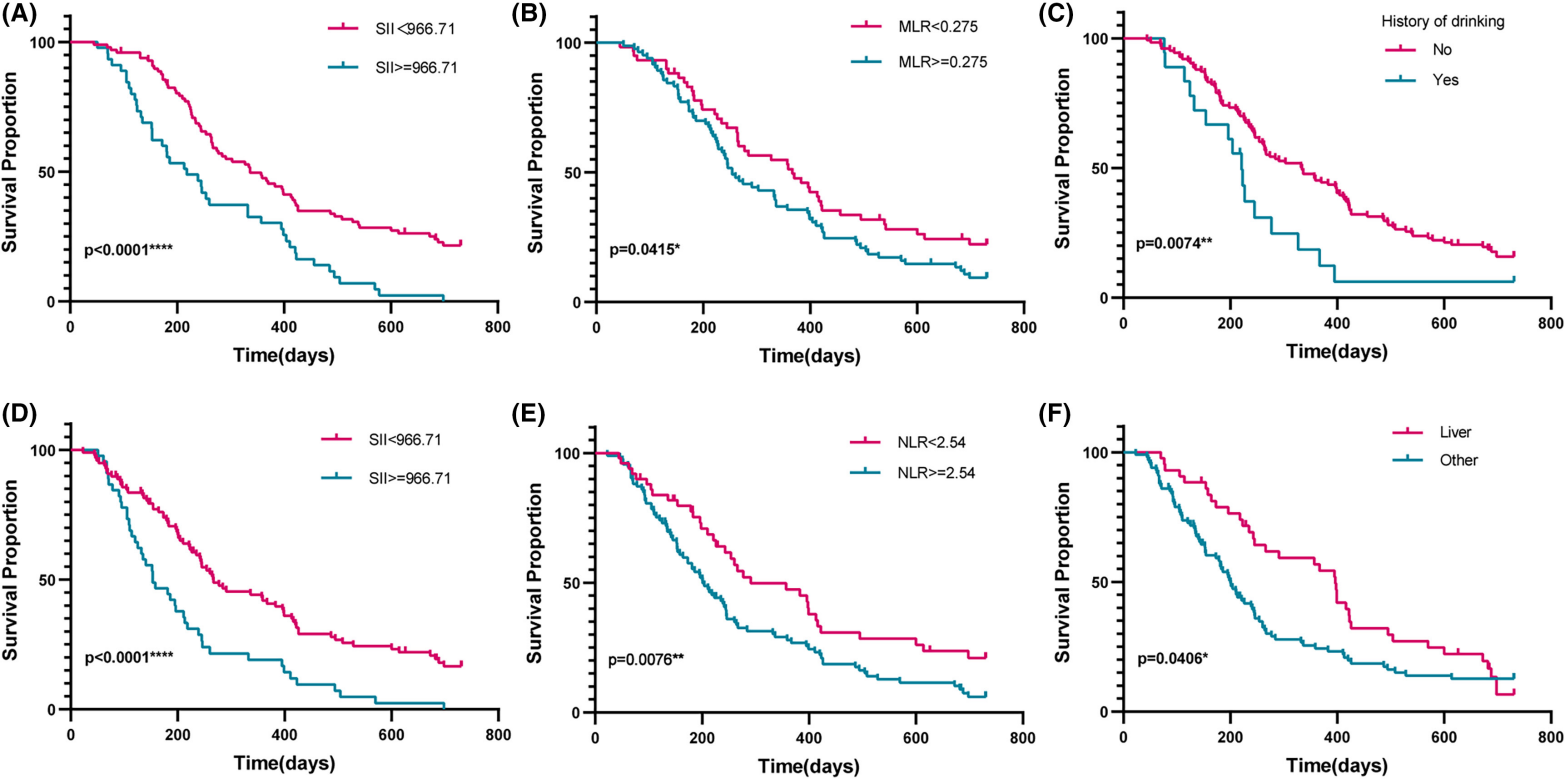
Kaplan–Meier curve and log‐rank test for OS based on SII (A), MLR (B) and history of drinking (C), PFS analysis in 143 pantients based on SII (D), NLR (E) and metastasis sites (F), *p* < 0.05 is considered statistically significant.

The Kaplan–Meier curve and log‐rank test were used to assess the prognostic value of age, sex, primary site, metastasis sites, baseline CA19‐9, baseline CEA, baseline SII, baseline MLR, baseline NLR, BMI, history of diabetes, history of smoking and history of drinking for predicting OS and PFS. Our findings suggested that higher baseline SII (≥ 966.71), higher baseline MLR (≥0.275) and drinking was correlated with poor OS prognosis (Figure 3A–C); higher baseline SII (≥ 966.71), higher baseline NLR (≥2.54) and liver metastases were associated with worse PFS (Figure 3D–F).

**FIGURE 4:**
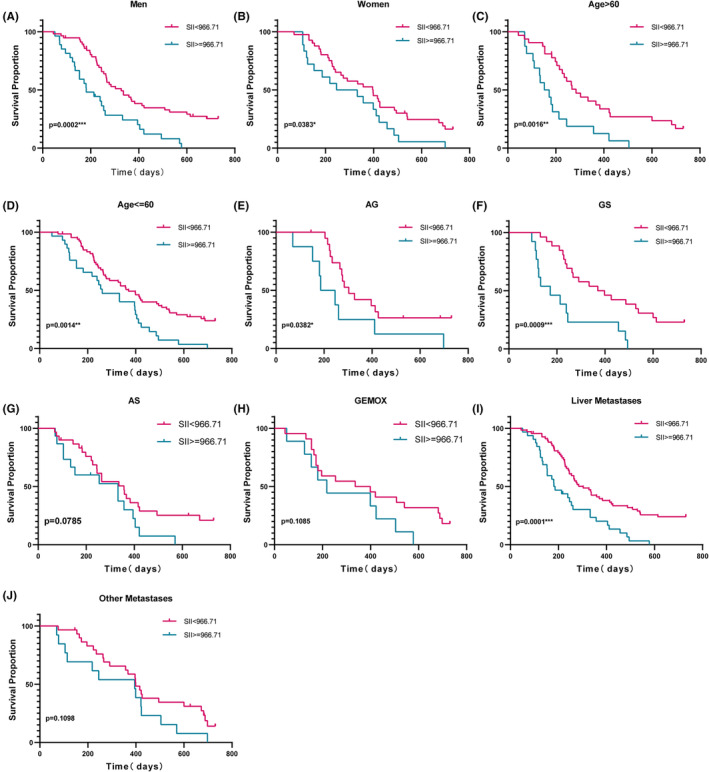
Correlation of SII with OS in sex (A, B), age (C, D), chemotherapy regimen (E–H) and metastasis sites (I, J) subtypes of metastatic pancreatic cancer receiving chemotherapy, *p* < 0.05 is considered statistically significant.

Then subgroup analyses based on age, gender, metastatic site, chemotherapy regimen, BMI, and primary site were carried out. Kaplan–Meier method analyses were carried out to determine whether these clinical factors affected the predictive ability of SII to predict OS in patients. Final, subgroup analysis showed that OS of men (*p* = 0.0002) (Figure 4A), women (*p* = 0.0383) (Figure 4B), age >60 (*p* = 0.0016) (Figure 4C), age ≤60 (*p* = 0.0014) (Figure 4D), AG regimen (*p* = 0.0382) (Figure 4E), GS regimen (*p* = 0.009) (Figure 4F) and liver metastases (*p* = 0.0001) (Figure 4I) were worse survival situation for those with SII ≥966.71, the above was statistically significant. OS of AS regimen (*p* = 0.0785) (Figure 4G), GEMOX regimen (*p* = 0.1085) (Figure 4H) and other metastases (*p* = 0.1098) (Figure 4J) were no statistically linked to SII. The stratified analysis's findings demonstrated that, in individuals with metastatic pancreatic cancer, the substantial connection between SII and OS was unaffected by age or gender.

### 3.3 The development of SII‐based nomograms for OS and PFS

**FIGURE 5:**
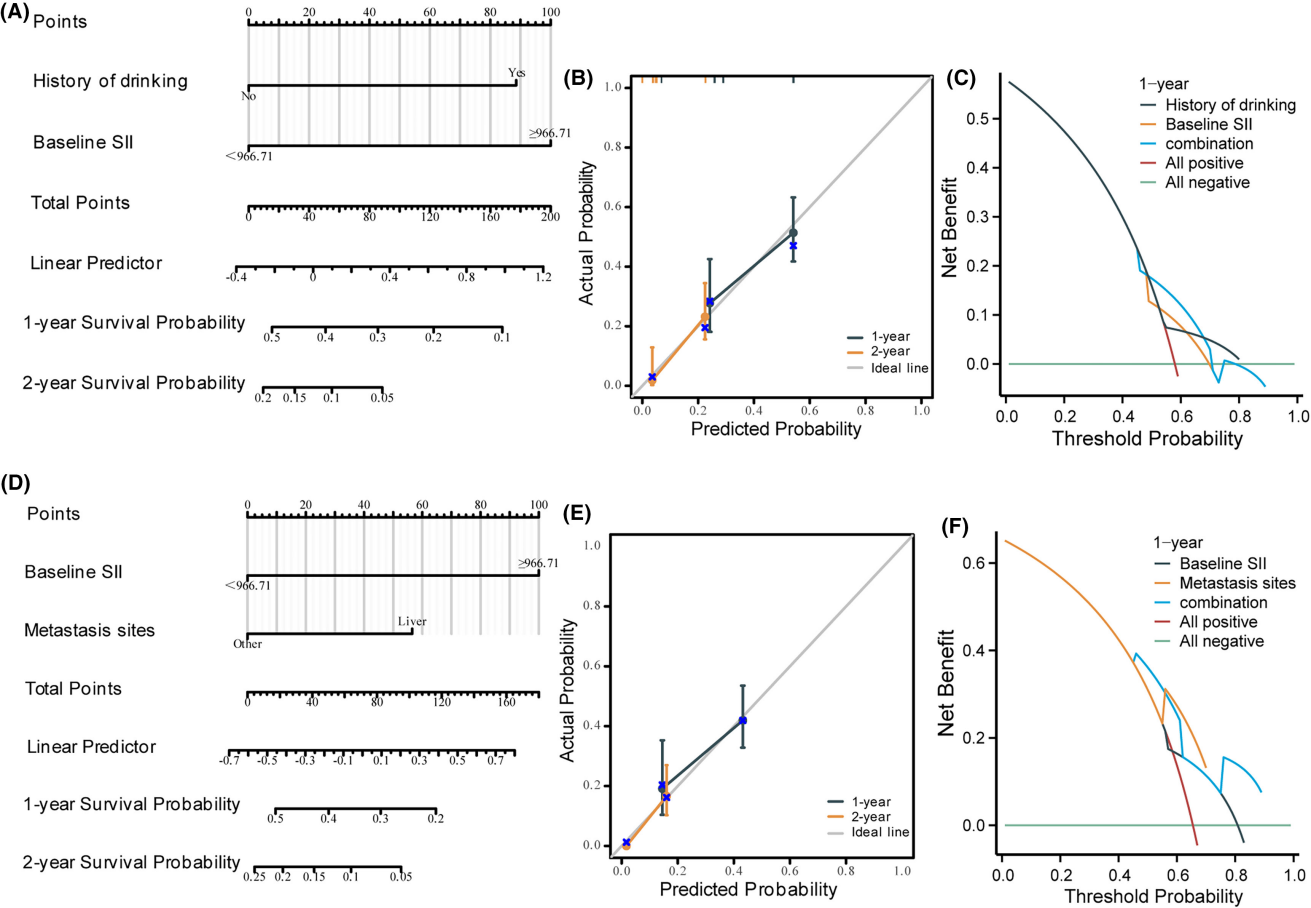
Developed nomogram to visualize 1‐year and 2‐year OS (A) and PFS (D); the 1‐year and 2‐year calibration curve for the prediction of OS (B) and PFS (E) model; 1‐year DCA for the prediction of OS (C) and PFS (F) model.

Univariate Cox regression analysis revealed that drinking, baseline SII, baseline MLR and baseline NLR were connected to OS, baseline SII and history of drinking were found to be independent risk factors for OS (Table 2) by multivariate Cox analysis. A unique nomogram (Figure 5A) was constructed based on these two independent prognostic indicators to visually forecast the 1‐ and 2‐year OS of the included patients. The calibration curves (Figure 5B) demonstrated a good degree of consistency between the actual probability and the anticipated possibilities of the model, while the 1‐year DCA curves (Figure 5C) assessed the nomogram's clinical efficacy in this experiment.

**TABLE 2:** Univariable and multivariable Cox regression of clinical risk factor of OS in all patients.

Characteristics	Total(N)	Univariate analysis		Multivariate analysis	
		Hazard ratio (95% CI)	*p* value	Hazard ratio (95% CI)	*p* value
Age	143				
≤60	95	Reference			
>60	48	1.433 (0.980–2.096)	0.063		
Sex	143				
Women	59	Reference			
Men	84	1.022 (0.710–1.473)	0.905		
Chemotherapy regimens	143				
AS	45	Reference			
AG	28	0.890 (0.524–1.513)	0.667		
GS	39	0.924 (0.576–1.483)	0.743		
GEMOX	31	0.926 (0.561–1.529)	0.765		
Primary site	143				
Head	47	Reference			
Neck	8	1.556 (0.893–2.711)	0.119		
Body	21	2.376 (0.988–5.712)	0.053		
Tail	34	1.466 (0.755–2.847)	0.894		
Head, Body	4	1.608 (0.892–2.899)	0.114		
Neck, Body	2	3.173 (1.069–9.423)	0.038		
Body, Tail	27	0.579 (0.077–4.338)	0.595		
Metastasis sites	143				
Liver	100	Reference			
Other	43	0.841 (0.569–1.243)	0.385		
Baseline CA19‐9	143				
<37	12	Reference			
≥37	127	0.826 (0.431–1.583)	0.565		
Unknow	4	0.874 (0.277–2.755)	0.818		
Baseline CEA	143				
<5	55	Reference			
≥5	81	1.391 (0.946–2.047)	0.094		
Unknow	7	1.321 (0.561–3.112)	0.525		
Baseline SII	143				
≥966.71	98	Reference		Reference	
<966.71	45	0.452 (0.309–0.662)	< 0.001	0.523 (0.333–0.820)	**0.005**
Baseline MLR	143				
≥0.275	59	Reference		Reference	
<0.275	84	0.679 (0.467–0.987)	0.042	1.050 (0.660–1.670)	0.836
Baseline NLR	143				
≥2.54	50	Reference		Reference	
<2.54	93	0.548 (0.368–0.817)	0.003	0.725 (0.437–1.204)	0.214
BMI	143				
<18.5	12	Reference			
18.5–24.9	98	0.958 (0.259–3.541)	0.948		
25.0–30.0	28	1.206 (0.381–3.822)	0.750		
>30	5	1.776 (0.535–5.890)	0.348		
History of diabetes	143				
No	126	Reference			
Yes	17	0.826 (0.464–1.471)	0.516		
History of smoking	143				
No	115	Reference			
Yes	28	1.495 (0.952–2.347)	0.080		
History of drinking	143				
No	125	Reference		Reference	
Yes	18	2.046 (1.196–3.499)	0.009	1.968 (1.149–3.370)	**0.014**

Abbreviations: AG, nab‐paclitaxel and gemcitabine; AS, Nab‐paclitaxel and S‐1; BMI, body mass index; CA19‐9, carbohydrate antigen19‐9; CEA, carcinoembryonic antigen; CI; confidence interval; GEMOX, gemcitabine and oxaliplatin; GS, gemcitabine plus S‐1; MLR, monocyte‐to‐lymphocyte ratio; NLR, neutrophil‐to‐lymphocyte ratio; SII, systemic immune inflammation index.

The bolded numbers (*p* < 0.05) are super significant findings from this study.

Meanwhile, baseline SII and metastasis sites were independent prognostic indices for PFS (Table 3) in this analysis. We also developed a nomogram (Figure 5D) of the predicted PFS based on the baseline SII and metastasis sites; similar to this, the calibration curves and DCA were used to assess the nomogram's clinical efficacy and predictive capability. A robust connection was seen between observation and prediction in the 1‐ and 2‐year calibration curves (Figure 5E). The nomogram was therapeutically helpful, according to the 1‐year DCA (Figure 5F).

**TABLE 3:** Univariable and multivariable Cox regression of clinical risk factor of PFS in all patients.

Characteristics	Total (*N*)	Univariate analysis		Multivariate analysis	
		Hazard ratio (95% CI)	*p* value	Hazard ratio (95% CI)	*p* value
Age	143				
≤60	95	Reference			
>60	48	1.388 (0.949–2.030)	0.091		
Sex	143				
Women	59	Reference			
Men	84	0.999 (0.693–1.440)	0.996		
Chemotherapy regimens	143				
AS	45	Reference			
AG	28	0.769 (0.451–1.310)	0.333		
GS	39	0.942 (0.587–1.514)	0.806		
GEMOX	31	0.881 (0.533–1.457)	0.623		
Primary site	143				
Head	47	Reference			
Neck	8	1.354 (0.778–2.357)	0.283		
Body	21	1.744 (0.727–4.184)	0.213		
Tail	34	1.235 (0.635–2.400)	0.534		
Head, Body	4	1.379 (0.765–2.487)	0.285		
Neck, Body	2	2.036 (0.687–6.036)	0.200		
Body, Tail	27	0.536 (0.071–4.022)	0.544		
Metastasis sites	143				
Liver	100	Reference		Reference	
Other	43	0.667 (0.451–0.988)	0.043	0.630 (0.422–0.940)	**0.024**
Baseline CA19‐9	143				
≥37	127	Reference			
<37	12	0.797 (0.416–1.527)	0.494		
Unknow	4	0.703 (0.223–2.219)	0.548		
Baseline CEA	143				
<5	55	Reference			
≥5	81	1.286 (0.875–1.890)	0.201		
Unknow	7	1.011 (0.429–2.384)	0.979		
Baseline SII	143				
≥966.71	45	Reference		Reference	
<966.71	98	0.451 (0.307–0.661)	< 0.001	0.466 (0.298–0.728)	**< 0.001**
Baseline MLR	143				
≥0.275	84	Reference			
<0.275	59	0.737 (0.507–1.071)	0.109		
Baseline NLR	143				
≥2.54	93	Reference		Reference	
<2.54	50	0.585 (0.393–0.870)	0.008	0.841 (0.531–1.334)	0.463
BMI	143				
<18.5	12	Reference			
18.5–24.9	98	0.846 (0.228–3.145)	0.803		
25.0–30.0	28	1.036 (0.325–3.299)	0.953		
>30	5	1.227 (0.369–4.082)	0.739		
History of diabetes	143				
No	126	Reference			
Yes	17	0.811 (0.455–1.444)	0.477		
History of smoking	143				
No	115	Reference			
Yes	28	1.297 (0.828–2.031)	0.257		
History of drinking	143				
No	125	Reference			
Yes	18	1.544 (0.906–2.631)	0.110		

Abbreviations: AG, nab‐paclitaxel and gemcitabine; AS, Nab‐paclitaxel and S‐1; BMI, body mass index; CA19‐9, carbohydrate antigen19‐9; CEA, carcinoembryonic antigen; CI, confidence interval; GEMOX, gemcitabine and oxaliplatin, GEMOX; GS, gemcitabine plus S‐1; MLR, monocyte‐to‐lymphocyte ratio; NLR, neutrophil‐to‐lymphocyte ratio; SII, systemic immune inflammation index.

The bolded numbers (*p* < 0.05) are super significant findings from this study.

### 3.4 Efficacy and adverse events of chemotherapy regimens

In this study, the mOS rate for 143 patients was 9.7 months. The mOS rates for the AG, AS, GEMOX and GS regimens were 9.2 months, 11.1 months, 11.2 months and 8.9 months, respectively. The study had 143 patients, of whom 45 (31%) received to the AS regimen, 28 (20%) to the AG regimen, 39 (27%) to the GS regimen, and 31 (22%) to the GEMOX regimen. According to the study's findings, 143 individuals had a mOS of 9.7 months. The median progression‐free survival for 143 patients was 8 months, whereas the mOS for the AS, AG, GS and GEMOX regimens was 11.1 months, 9.2 months, 8.9 months, and 11.2 months, respectively.

Table S1 illustrates that the ORR for 143 patients was 6.3%, while the corresponding values for AS, AG, GS and GEMOX were 6.7%, 14.3%, 5.1%, and 0%. The DCR for 143 patients was 49.0%, compared with 40%, 57.1%, 46.2% and 58.1% for AS, AG, GS and GEMOX, respectively (Table S1).

In our retrospective cohort research, the incidence of myelosuppressive adverse reactions was higher than that of other adverse reactions, as Table S2 illustrates. For AS, AG, GS and GEMOX, the corresponding incidence of Grade III–IV adverse events was 11.1%, 7.1%, 2.6% and 6.5% (Table S2). It was evident that the AS regimen had the lowest DCR and the largest prevalence of grade III–IV adverse events.

## 4 DISCUSSION

According to data released by the American Cancer Society, metastatic pancreatic cancer has a low survival rate and ranks third overall in terms of causes of death.^22^ Currently, the most advanced treatment option for metastatic pancreatic cancer is multi‐agent combination chemotherapy. Metastatic pancreatic cancer^23^, ^24^, ^25^, ^26^ is known for its extreme heterogeneity and high aggressiveness, which can result in poor individual treatment outcomes and different treatment outcomes for different TMEs. Our clinical work has also shown that chemotherapy affects patients differently even if they have the same stage and metastatic site, thus based on the discovery of independent prognostic markers, clinically significant nomograms can be established to estimate the personalized survival of patients. The FOLFIRINOX and AG regimens have been approved as first‐line therapies for metastatic pancreatic cancer by the National Comprehensive Cancer Network in the 10 years following the results of the PRODIGE4/ACCORD 11 and MPACT investigations. Our analysis of patient records from the Harbin Medical University Cancer Hospital, there are relatively few FOLFIRINOX regimens, this is likely because of the high frequency of toxicities and side effects associated with the FOLFIRINOX regimen and the fact that, consequently, we included individuals receiving AS, AG, GS, and GEMOX for metastatic pancreatic cancer. This study's main goal is to gather real‐world patient data, organize it, and then use statistical analysis to determine the independent prognostic factors of patients with metastatic pancreatic cancer receiving chemotherapy with regimens that include AS, AG, GS and GEMOX. Additionally, the study aims to find effective biomarkers and build visual nomogram models that will enable timely prediction of the patients' survival probability, which can serve as a guide for clinical staff involved in patient care.

Baseline SII and history of drinking, baseline SII and tumor metastatic site were independent prognostic indicators for OS and PFS, respectively, and had predictive value for patient survival, according to our multivariate Cox proportional risk model analysis. Previous studies by other investigators have shown that peripheral blood routine index^27^, ^28^, ^29^, ^30^ is one of the important indicators for assessing TME, tumor inflammatory response, tumor drug resistance, and tumor progression. Peripheral blood cells are closely related to inflammatory response,^31^ and tumor development and tumor efficacy response to drugs are inextricably linked to inflammatory response. Inflammatory responses are involved to some extent in tumor formation and progression during tumor development,^17^ white blood cell count, neutrophil count, and lymphocyte count in routine peripheral blood indicators can reflect the degree of inflammatory response. Increased neutrophil count^32^ may indicate the presence of an inflammatory response in the body, while changes in lymphocyte count^33^, ^34^, ^35^ may be related to immune system activity. The process of tumor cell proliferation, invasion, and spread is referred to as tumor progression, increased tumor angiogenesis and invasive ability may be associated with higher neutrophil and platelet counts in regular peripheral blood indicators.^36^ Elevated neutrophils and elevated platelet counts in regular peripheral blood indicators may be associated with increased tumor angiogenesis and invasiveness. Peripheral blood cells are therefore vital to the survival and progression of malignancies. SII is a bioinflammatory index that is used to visually assess the immune‐inflammatory status of tumor patients. It is a straightforward, cost‐effective biomarker that can respond to a body's comprehensive assessment of the immune‐inflammatory status. SII is calculated by combining lymphocyte count, neutrophil count, and platelet count. SII is a good indicator of the level of immune‐inflammatory response in the body of a tumor patient, according to several studies,^19^, ^37^ SII is frequently used to help determine prognostic risk in tumor patients. Elevated SII values may indicate increased immune system activity and heightened inflammatory response, which is typically associated with a poor prognosis, including shorter survival and higher rates of disease recurrence. By incorporating 164 patients with pancreatic cancer who had resections and a cutoff SII value of 563, Chen et al.^37^ found that the high SII group was a separate risk factor for OS, individuals with a worse prognosis and a lower SII had extensive recurrence, only TNM staging was revealed to be an independent predictive predictor for patient recurrence, the researchers speculate that this could be because tumor development and peripheral immune status are tightly related. Widely used in the investigation of various cancers, SII has been linked to poorer overall survival, tumor‐specific survival, and recurrence‐free survival according to Li et al.'s analysis of 10 analyses involving 7087 patients with bladder cancer,^38^ furthermore, there was a correlation seen between larger preoperative SII and poorer tumor differentiation, higher tumor stage, involvement of lymph nodes, and tumor size ≥3 cm. The findings of bladder cancer were generally consistent with the meta‐analysis of a different study^39^ on colorectal cancer. This demonstrates that SII is a very reliable predictor with good predictive value in malignant tumors.

Alcohol intake^40^ has long been linked to a higher risk of pancreatic cancer and chronic pancreatitis, according to several studies. One possible mechanism^41^, ^42^ of alcohol‐related carcinogenesis is the body's conversion of alcohol to acetaldehyde, reactive oxygen species, and nitrogen synthesis. It might directly harm cells or cause mechanisms like oxidative stress that result in lipid peroxidation, which lessens the cell's capacity to scavenge free radicals and causes cellular damage and carcinogenesis, or it might affect the metabolism of folate, which modifies DNA methylation and consequently regulates genes that may be involved in the development of cancer. Additionally, drinking alcohol may weaken immune surveillance, which increases the risk of cancer growth and metastasis.^43^ According to our research, patients with a history of drinking have a worse prognosis, and history of drinking is an independent risk factor for OS. Lu et al.^44^ found that Western and heavy drinking may raise the risk of pancreatic cancer, healthy and light‐moderate alcohol consumption may lower the risk of pancreatic cancer in a meta‐analysis, which was confirmed by another study.^45^ Research on alcohol and cancer in clinical, epidemiologic, and experimental contexts is still scarce, despite the significance of alcohol in human carcinogenesis. Controlling alcohol misuse, however, continues to be a key objective of cancer control due to the linear dose–response association between alcohol intake and cancer risk.

The liver is the most typical place where pancreatic cancer metastasizes, but it can also spread to other locations like the peritoneum, lungs, bones and lymph nodes. The majority of patients in this trial had liver metastases; and liver metastases had a statistically significant worse prognosis for PFS than other metastases, according to the Kaplan–Meier curve. Yao et al.^46^ looked through the SEER database for 11,287 patients, 601 (5.3%) of whom had lung metastases at the time of PC diagnosis. Patients with lung metastases alone would have a longer OS than those without lung metastases alone, and liver metastasis, chemotherapy were found to be independent prognostic variables for OS and cancer‐specific survival, and bone metastasis was an independent predictor of cancer‐specific survival. Multiple distant metastases and a poor outcome for chemotherapy recipients were also shown in another investigation,^47^ in particular, a total of 2072 cases were found in the SEER database for another study which focused on the prognostic investigation of pancreatic cancer with bone metastases, the analysis conducted revealed that factors such as age, pathology type, chemotherapy administration status, liver metastasis, and lung metastasis were independent predictors of OS, individuals with chemotherapy had a better prognosis, while those with lung and liver metastases had a worse prognosis. In our study, as long as they had liver metastases, the 143 pancreatic cancer patients included in this study were classified as having liver metastases; other metastases from other sites but not liver metastases were classified as belonging to the other metastasis group. In the comprehensive analysis, patients with multisite distant metastases demonstrated a significantly worse prognosis, furthermore, patients with liver metastases of pancreatic cancer may exhibit a more unfavorable prognosis in comparison to other metastases. 

In order to illustrate the analysis, our study effectively created nomograms about metastatic pancreatic cancer patients treated with AS, AG, GS, and GEMOX regimens using the R‐Project for the independent prognostic factors. Studies demonstrating constructed nomograms, constructed using baseline clinical factors that are readily available prior to chemotherapy, have shown good performance and are very convenient in assisting clinicians in providing individualized management for patients. Zhang et al.^21^ study included 235 patients screened for pancreatic cancer liver metastases from the SEER database, 167 patients for the training set, 68 patients for the internal validation set as well as retrospectively collected pancreatic cancer patient data. By using Cox regression analysis, it was discovered independent predictors, based on the AUC of ROC analysis, calibration plot and DCA, they concluded that the risk and prognostic model of pancreatic cancer bone metastasis had good performance and the nomogram could accurately predict the risk and prognostic factors of pancreatic cancer bone metastasis. Deng et al.^48^ enrolled patients with advanced and metastatic pancreatic cancer who received first‐line chemotherapy, a validation cohort for this study was subsequently assembled from a prospective study of 236 patients with advanced and metastatic pancreatic cancer receiving first‐line chemotherapy, this led to the development of an accurate and well‐performing nomogram model based on independent prognostic factors, which will help doctors with the initial survival assessment at the time of diagnosis. Helpful nomograms of research regarding pancreatic cancer patients' survival prognosis are a lot. But our study is the first to model the prediction of OS and PFS using the SII index for nomograms for patients with metastatic pancreatic cancer treated with AS, AG, GS and GEMOX regimens, based on our understanding of real‐world studies, and the model performed well in predicting survival. Therefore, it is anticipated that our study will be a useful tool to support clinical decision making.

This study does have certain drawbacks, though. First, because it is a retrospective, single‐center cohort study, biases in information and selection may exist. Further validation of our findings through prospective multicenter trials is necessary to lower these bias mistakes and enhance the trustworthiness of the results. To properly apply the measurements to the individualized care of each patient, we also need to record the measures' dynamic changes, because pancreatic cancer is such a highly variable disease, patients' circumstances and reactions to therapy might fluctuate over time. We will therefore be able to comprehend patient illness development and treatment outcomes better if we monitor and record dynamic changes in markers. In conclusion, even with a few drawbacks, this study provides us with a promising new predictive model for nomograms that can be applied to clinical practice. Through further research and validation, we can further improve the accuracy and reliability of the model and use it to assist clinical decision‐making and individualized management.

## 5 CONCLUSION

In this study, we found that the higher the SII level (≥ 966.71), the lower the survival rate of metastatic pancreatic cancer patients receiving systemic chemotherapy. Based on this finding, we developed meaningful nomogram models for predicting patients' OS and PFS. With models, we visualized the importance of SII in predicting the survival of metastatic pancreatic cancer patients receiving systemic chemotherapy.

## AUTHOR CONTRIBUTIONS

**Yanan Sun:** Conceptualization (equal); formal analysis (lead); methodology (lead); writing – original draft (equal). **Jiahe Hu:** Writing – original draft (equal). **Rongfang Wang:** Data curation (equal). **Xinlian Du:** Data curation (equal). **Xiaoling Zhang:** Data curation (equal). **Jiaoting E:** Data curation (equal). **Shaoyue Zheng:** Data curation (equal); investigation (equal). **Yuxin Zhou:** Data curation (equal); investigation (equal). **Ruishu Mou:** Data curation (equal); investigation (equal). **Xuedong Li:** Data curation (equal); investigation (equal). **Hanbo Zhang:** Data curation (equal); investigation (equal). **Ying Xu:** Data curation (equal); investigation (equal). **Yuan Liao:** Writing – review and editing (supporting). **Wenjie Jiang:** Writing – review and editing (supporting). **Lijia Liu:** Writing – review and editing (supporting). **Ruitao Wang:** Writing – review and editing (equal). **Jiuxin Zhu:** Conceptualization (equal). **Rui Xie:** Conceptualization (equal); funding acquisition (lead); writing – review and editing (equal).

## FUNDING INFORMATION

This work were supported by the National Natural Science Foundation of China (Grant No. 82073301) and The Haiyan Foundation of the Harbin Medical University Cancer Hospital (JJMS2021‐03 : JJMS2021‐22 : JJMS2021‐23).

## CONFLICT OF INTEREST STATEMENT

The authors have no relevant financial or non‐financial interests to disclose.

## ETHICS STATEMENT

Not applicable.

## CONSENT TO PUBLISH

Each author agreed to publish this paper.

## CONSENT TO PARTICIPATE

This study was a clinical retrospective study, so it was not required.

## Supporting information


Table S1:

Table S2:

## Data Availability

The datasets generated during and/or analyzed during the current study are available from the corresponding author on reasonable request.
