# Supplementary material for: Meaningful nomograms based on systemic immune inflammation index predicted survival in metastatic pancreatic cancer patients receiving chemotherapy
Source: Cancer Med. 2024 Jul 10;13(13):e7453. doi: 10.1002/cam4.7453 (PMC11236459; doi:10.1002/cam4.7453)
Supplement: Supplementary file 1 — Table S1: [file CAM4-13-e7453-s001.docx]

**Table S1** The diference of tumor response between four regimens, n (%).

| Tumor response | AS（n=45） | AG（n=28） | GS（n=39） | GEMOX（n=31） |
| --- | --- | --- | --- | --- |
| Complete response (CR) | 0（0） | 1（3.6） | 0（0） | 0（0） |
| Partial response (PR) | 3（6.7） | 3（10.7） | 2（5.1） | 0（0） |
| Stable disease (SD) | 18（40） | 16（57.1） | 18（46.2） | 18（46.2） |
| Progressive disease (PD) | 12（26.7） | 5（17.9） | 15（38.5） | 5（12.8） |
| Objective response rate(%) | 3（6.7） | 4（14.3） | 2（5.1） | 0（0） |
| Disease control rate (CR+PR+SD) | 18（40） | 16（57.1） | 18（46.2） | 18（46.2） |

**Abbreviations:** Nab-paclitaxel and S-1, AS; nab-paclitaxel and gemcitabine, AG; gemcitabine plus S-1, GS; gemcitabine and oxaliplatin, GEMOX.
