# Supplementary material for: Meaningful nomograms based on systemic immune inflammation index predicted survival in metastatic pancreatic cancer patients receiving chemotherapy
Source: Cancer Med. 2024 Jul 10;13(13):e7453. doi: 10.1002/cam4.7453 (PMC11236459; doi:10.1002/cam4.7453)
Supplement: Supplementary file 2 — Table S2: [file CAM4-13-e7453-s002.docx]

**Table S2** Adverse events in patients.

|  | AS（n=45） | AG（n=28） | GS（n=39） | GEMOX（n=31） |
| --- | --- | --- | --- | --- |
| Myelosuppression | 14（31.1） | 14（50） | 13（33.3） | 10（32.3） |
| Gastrointestinal adverse effects | 6（13.3） | 4（14.3） | 11（28.2） | 10（32.3） |
| Fatigue | 0（0） | 4（14.3） | 5（12.8） | 3（9.7） |
| Others | 1（2.2） | 0（0） | 1（2.6） | 0（0） |
| Grade III-IV adverse events | 5（11.1） | 2（7.1） | 1（2.6） | 2（6.5） |

**Abbreviations:** Nab-paclitaxel and S-1, AS; nab-paclitaxel and gemcitabine, AG; gemcitabine plus S-1, GS; gemcitabine and oxaliplatin, GEMOX.
